# A selective inhibitor of the Rho kinase pathway, Y-27632, and its influence on wound healing in the corneal stroma

**Published:** 2012-06-27

**Authors:** Mayumi Yamamoto, Andrew J. Quantock, Robert D. Young, Naoki Okumura, Morio Ueno, Yuji Sakamoto, Shigeru Kinoshita, Noriko Koizumi

**Affiliations:** 1 Faculty of Life and Medical Sciences, Doshisha University, Kyotanabe, Kyoto, Japan; 2 School of Optometry and Vision Sciences, Cardiff University, Wales, UK; 3 Department of Ophthalmology, Kyoto Prefectural University of Medicine, Kyoto, Japan

## Abstract

**Purpose:**

Our study examined the effect of a selective Rho kinase inhibitor, Y-27632, on corneal wound healing and potential stromal scarring after superficial keratectomy.

**Methods:**

Rabbit keratocytes were induced into myofibroblasts by transforming growth factor β1 (TGFβ1) either with or without Y-27632. Then α-smooth muscle actin (α-SMA) was examined by immunohistochemistry and western blotting, and the contractility of the seeded collagen gels was measured. Y-27632 eye drops (or vehicle only) were administered to eyes after a superficial keratectomy, and the tissue was examined by immunohistochemistry for α-SMA, collagen types I, II, and III, and keratan sulfate. Electron microscopy was conducted with and without histochemical contrasting of sulfated proteoglycans.

**Results:**

Spindle-like cells in culture constituted 99.5±1.1% with TGFβ1 stimulation, but 3.5±1.0% after TGFβ1 and Y-27632 treatment (p<0.01, n=6). α-SMA was seen in 4% of TGFβ1-treated cells, but in only 0.3% of cells with Y-27632 added (p<0.01, n=6), which was confirmed by western blotting. Y-27632 also inhibited the TGFβ1-induced contraction of seeded collagen gels. After superficial keratectomies, collagen type I and keratan sulfate were unchanged by Y-27632 application. Collagen type II was not detected in Y-27632 or vehicle-only corneas. With Y-27632 treatment, α-SMA expression increased and the collagen type III signal became in the weaker subepithelial area. Interestingly, bundles of aligned and uniformly spaced collagen fibrils were more prevalent in keratocytes in Y-27632-treated corneas, which is reminiscent of fibripositor-like structures that have been proposed as a mechanism of matrix deposition in embryonic connective tissues.

**Conclusions:**

Y-27632 inhibits keratocyte-to-myofibroblast transition, and its topical application after a superficial lamellar keratectomy elicits an altered wound healing response, with evidence of an embryonic-type deposition of collagen fibrils.

## Introduction

Keratocytes are quiescent in mature healthy cornea, but after an injury or surgery, they differentiate into myofibroblasts and migrate to the wound site [1-4]. This phenotypic transformation is identified by the presence of microfilament bundles or stress fibers in myofibroblasts, which are associated with 1) the expression of α-smooth muscle actin (α-SMA) and 2) the spindle-like morphology of myofibroblasts compared to dendritic keratocytes [5-8]. The expression of α-SMA during corneal wound healing is important for cell migration and wound contraction [9]. However, the presence of excess numbers of myofibroblasts in wounded tissue is undesirable because of the risk of fibrotic scar formation. Thus, investigations into possible regulators of keratocyte-to-myofibroblast transformation offer significant scope for future intervention strategies for modulating wound healing in the cornea.

A key factor in the keratocyte-to-myofibroblast transition is transforming growth factor β (TGFβ) [10-12]. *TGFβ1* mRNA and protein are present in the corneal epithelium and corneal stroma, and both paracrine and autocrine TGFβ1 response pathways are involved in the induction of keratocyte transformation [13-16]. Multiple signaling cascades are activated when TGFβ binds to its cognate receptor. These include Smad [17], RhoA-related signals [18], mitogen-activated protein kinase (MAPK)-Erk-1 and −2 [19], stress kinases (i.e., c-Jun N-terminal kinase [JNK]) [20,21], p38 mitogen-actiated protein kinase (p38MAPK) [22,23], phosphatase 2A [24], and phosphoinositide 3-kinase/AKT (PI3K/AKT) [25,26]. The pathways involved in cellular differentiation or transformation are Smad, Rho proteins, and PI3-kinase.

It is known that assembly and organization of actomyosin filaments to transform keratocytes into myofibroblasts are regulated by Rho GTPases. One of the downstream effectors of Rho is Rho-associated coiled-coil containing protein kinase (ROCK), which is a serine/threonin protein kinase that contains an NH_2_-terminal catalytic kinase domain and plays an important role in the activation of actin/myosin interactions and smooth muscle cell contraction by maintaining the activity of myosin light chain kinase (MLCK). Previous investigations showed that ROCK inhibitor (Y-27632) inhibited keratocyte fibrosis in vitro [27]. Other research has shown that Y-27632 has potential beneficial effects via its inhibition of apoptosis [28] and invasive carcinoma [29], the stimulation of cell proliferation in primate corneal endothelial cells [30], the suppression of kidney fibrosis [31], and the regulation of cell differentiation in embryonic stem cells [32]. In the current study, we focus on the Rho signaling pathway, which we attempted to block using a selective Rho-associated coiled-coil containing protein kinase (ROCK) inhibitor, Y-27632 [33], both in vitro and in vivo to suppress the differentiation of keratocytes into myofibroblasts and modulate cell-driven wound healing.

## Methods

Rabbit corneas and isolated cells were used as the model system for our study of wound healing [34,35].

### Cell culture

Rabbit corneas were incubated with 1.2 U/ml Dispase (Life Technologies Japan Ltd, Tokyo, Japan) for 1 h at 37 °C, after which the corneal epithelium and endothelium were removed by mechanical scraping. The stroma was then cut into small, approximately 1 cm^2^ pieces, which were incubated overnight at 37 °C in DMEM/F12 containing 1 mg/ml collagenaseA (Roche Diagnostics K.K., Tokyo, Japan) and 1% penicillin-streptomycin. After centrifugation at 440× g for 3 min, the cells were sub-cultured in serum-free medium (DMEM/F12 containing with 10 μg/ml insulin, 1 mM ascorbic acid, and 1% penicillin-streptomycin) for 48 h. They were then induced into myofibroblasts by TGFβ1 (3 ng/ml; R&D systems, Minneapolis, MN) with or without a 2 h pre-incubation with 10 μM Y-27632 (Wako, Osaka, Japan). After 48 h, cell phenotype was observed by phase contrast light microscopy (Leica CTR 4000; Leica Microsystems GmbH, Wetzlar, Hesse, Germany), and examined by immunofluorescence and western blotting for the myofibroblast marker α-SMA. To calculate the percentage of spindle-like cells, micrographs were taken at six different areas in each well. The total number of cells and the number of spindle-like cells was counted.

### Immunohistochemistry for α-SMA

Cells were fixed by immersion in 4% paraformaldehyde for 10 min, after which they were washed three times with phosphate-buffered saline (PBS), permeabilized with 0.5% Triton X-100, blocked with 1% bovine serum albumin (BSA) in PBS for 30 min at room temperature, and then incubated with α-SMA (1:400; Thermo Fisher Scientific K.K, Yokohama, Kanagawa, Japan) antibody or mouse immunoglobulin G 2a (IgG2a) isotype control for 2 h at room temperature. This was followed by incubation with AlexaFluor 488-conjugated secondary antibody (Invitrogen) in a 1:2000 dilution. Nuclei were counterstained with 4',6-diamidino-2-phenylindole (DAPI; Vector Laboratories Inc., Burlingame, CA).

### Western blotting

Cells were washed with PBS and extracted in lysis buffer (50 mM Tris-HCL, 5 mM EDTA, 0.15 M NaCl, 1% TritonX-100, pH 8.0) containing protease inhibitor and phosphate inhibitor. Lysed cells were centrifuged at 90× g for 10 min at 4 °C, after which the supernatant was collected and stored at −80 °C until required. Protein assay was performed using a BCA™ protein assay kit (Thermo Fisher Scientific) and protein concentration was measured at 562 nm. Equal amounts of protein were resolved by SDS–PAGE (4% to 12% tri-acetate mini gel; Invitrogen) and transferred to polyvinylidene difluoride membranes. The membranes were blocked with 1% skimmed milk dissolved in tris buffered saline Tween (TBS-T; 50mM Tris-HCl, 150 mM NaCl, 0.05% Tween-20), before incubation overnight at 4 °C with α-SMA (1:1,000) and β-actin (1:3,000) primary antibody diluted in 1% skimmed milk. After the blots were washed with TBS-T, they were incubated with horseradish peroxidase conjugated secondary IgG (GE Healthcare, Bucks, UK). The reacted proteins were revealed by an enhanced chemiluminescence system (GE Healthcare).

### Collagen gel contraction assays

Fibroblast-mediated gel contraction with or without Y-27632 was measured. Type I collagen gels (AteloCell®; Koken, Tokyo, Japan) were produced in the form of a viscous liquid as described previously [36] to achieve a final concentration of collagen of 1.9 mg/ml. These were seeded with keratocytes to a final cell density of 2×10^5^ cells/ml, after which 0.25 ml of the resultant mixture was added to a 48-multiwell plate coated with 1% BSA. This was incubated for 1 h at 37 °C to induce gelation. Serum-free medium was then added to each well for 48 h followed by the addition of 30 ng/ml TGFβ1, with or without 100 μM Y-27632. The area of the collagen gels was measured every 24 h for three days using ImageJ software.

### Surgical procedures

Four adult male rabbits (Japanese White) each weighing 2.5 kg to 3.0 kg underwent bilateral superficial keratectomies 7.5 mm in diameter and approximately 150 μm deep. At all times the animals were treated according to full ethical approval. A quarter turn with a BARRON radial vacuum trephine (Katena Products, Denville, NJ) was used to achieve approximately standard depth, with all surgeries conducted by the same surgeon. The keratectomy was achieved by a freehand lamellar dissection, and the thickness of the residual stromal bed was measured using a TOMEY ultrasonic pachymeter (Tomey Corporation, Nagoya, Japan). After surgery, topical antibacterial agent (0.3% ofloxacin eye drops) was applied. Postoperatively, Y-27632 (10 mM) eye drops were administered to the right eyes of all rabbits four times daily for three weeks, with vehicle only added to the left eyes, which acted as controls. Two non-operated rabbits also received this daily application of Y-27632 in one eye and vehicle in the other. Fluorescein staining was used to monitor epithelial healing.

### Immunohistochemistry for matrix components

After three weeks of eye drop treatment, the animals were euthanized and all 12 corneas (Y-27632-treated surgery group (n=4); vehicle-treated surgery group (n=4); Y-27632-treated non-surgery group (n=2); vehicle-treated non-surgery group (n=2) were excised, bisected,and half embedded in Optimal Cutting Temperature (OCT) compound; the other half was prepared for electron microscopy as described below. At room temperature, cryosections 8 µm thick were rehydrated with PBS for 3 min, fixed in 70% ethanol for 1 min, washed three times with PBS, and blocked with 5% goat serum (or 1% BSA with no fixation for the 5D4 group) in PBS for 30 min. Sections were incubated at room temperature for 2 h with antibodies to type I collagen (1:2,000; Sigma-Aldrich), type II collagen (1:1; provided by Prof. Victor Duance, School of Biosciences, Cardiff University), type III collagen (1:2,000; Sigma-Aldrich) and minimally pentasulfated keratan sulfate (5D4; 1:500; provided by Prof. Bruce Caterson, School of Biosciences, Cardiff University). The control sections were incubated with mouse IgG1 isotype. Sections were then labeled with Alexa Fluor 488 secondary antibody (1:2,000), mounted with the nuclear stain DAPI (Vectashield*™*) and analyzed using an Olympas BX61 (Olympus Corporation, Tokyo, Japan) microscope and F-View digital camera. Sections of rabbit ear cartilage were used as a positive control for collagen type II immunohistochemistry. The α-SMA immunostaining was performed on tissue sections using the protocol described above.

### Electron microscopy

Excised half corneas were cut into four equal sectors: two were prepared for an examination of cell and matrix morphology and two were prepared for proteoglycan visualization. For cellular examination, tissues were fixed in 2.5% glutaraldehyde and 2% paraformaldehyde in 0.1 M Sörensen buffer, pH 7.2–7.4 for 2 to 3 h at room temperature. Following several washes in the buffer and post-fixation with 1% aqueous osmium tetroxide, they were processed through 0.5% uranyl acetate to contrast collagen, dehydrated through an ascending ethanol series and embedded in Araldite resin (Agar Scientific, Cambridge, UK). For proteoglycan localization, tissues were immersed overnight in 2.5% glutaraldehyde in 25 mM sodium acetate buffer, 0.1 M MgCl_2_ containing 0.05% Cuprolinic Blue [37-39]. The next day, after washes in fixation buffer minus the blue dye and enhancement by three washes in aqueous 0.5% sodium tungstate, the tissues were dehydrated as before and embedded in Araldite resin. Semi-thin sections (1 µm thick) were stained with Toluidine blue for inspection at the light microscope level, while ultrathin sections (approximately 80 to 100 nm thick) were collected on uncoated copper grids for study by transmission electron microscopy. Sections on grids were stained with aqueous uranyl acetate and Reynolds’ lead citrate for matrix morphology, then uranyl acetate, and finally phosphotungstic acid for imaging proteoglycan-collagen associations. Specimens were examined using a transmission electron microscope (JEM1010; JEOL, Tokyo, Japan) equipped with a CCD camera (Gatan ORIUS SC1000; Gatan Inc., Pleasanton, CA).

## Results

**Figure 1:**
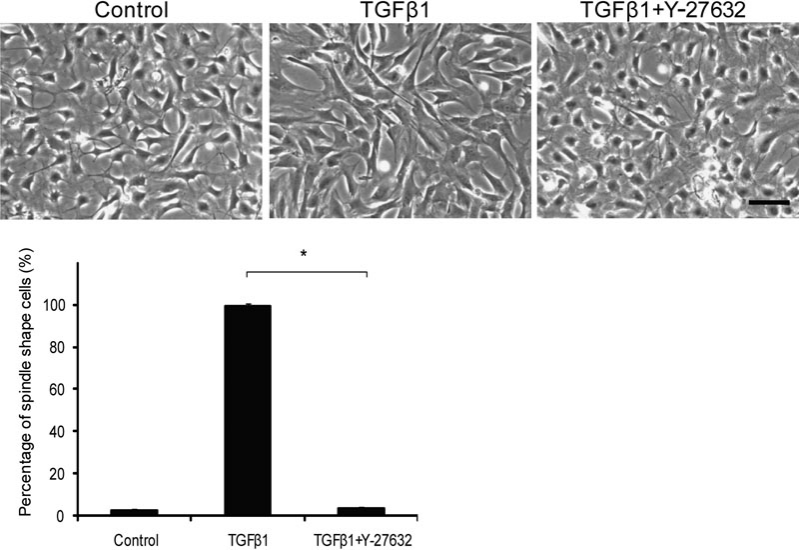
The effect of Y-27632 on keratocyte morphology. The percentage of spindle-like cells was 99.5±1.1% with TGFβ1 stimulation, but 3.5±1.0% in cells treated with TGFβ1 and Y-27632. Values are means±SEM (n=4). *p<0.01 (Student’s *t*-test). Scale bar: 100 μm.

**Figure 2:**
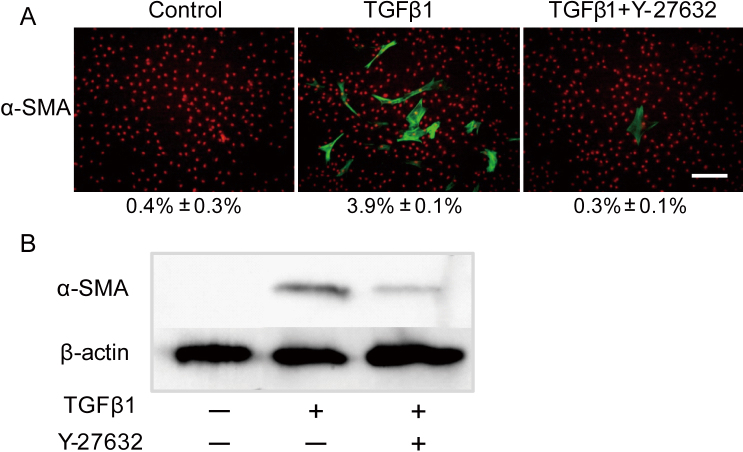
The effect of Y-27632 on the transformation of keratocytes. **A**: On immunohistochemistry α-SMA expression was seen in about 4% of cells in culture with TGFβ1 stimulation, but in only 0.3% of cells treated with TGFβ1 and Y-27632 (p<0.01, Student’s *t*-test). Values are means±SEM (n=6). Scale bar: 200 μm. **B**: Western blots revealed that the expression of α-SMA was significantly decreased in keratocytes treated with Y-27632, but not abolished.

**Figure 3:**
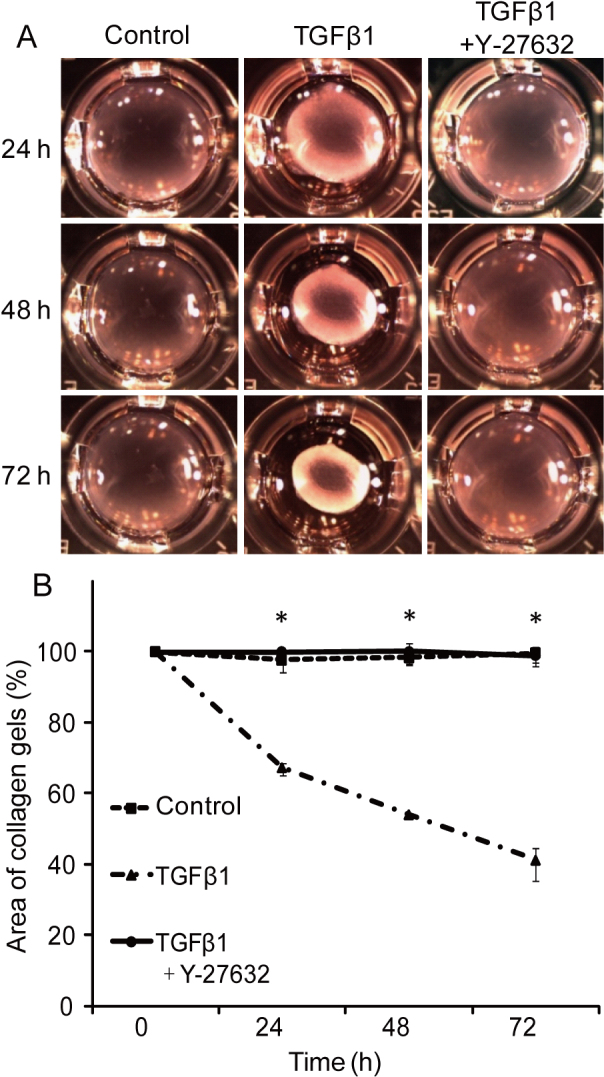
The effect of Y-27632 on fibroblast contractility. **A**: TGFβ1 induced collagen contraction over time. Y-27632-application significantly inhibited the contraction of fibroblast-seeded collagen gels. **B**: Statistical analysis of the area of collagen gels by ImageJ software. Values are means±SEM (n=3). *p<0.01 (Student’s *t*-test).

Keratocytes in cell culture were induced into myofibroblasts after 48 h of TGFβ1 stimulation. The percentage of spindle-like cells was 99.5±1.1% with TGFβ1 stimulation, but 3.5±1.0% in cells treated with TGFβ1 and Y-27632 (Figure 1). As a marker of myofibroblast phenotype, α-SMA expression was seen by immunohistochemistry in about 4% of cells in culture with TGFβ1 stimulation, but in only 0.3% of cells treated with TGFβ1 and Y-27632 (Figure 2A). Western blots revealed that the expression of α-SMA was significantly decreased, but not abolished, in keratocytes treated with TGFβ1 and Y-27632 compared with cells treated with TGFβ1 only (p<0.01, Student’s *t*-test; Figure 2B). To investigate whether or not the inhibition by Y-27632 of the TGFβ1-mediated phenotypic differentiation could influence the contractile ability of cells in vitro, keratocytes treated with TGFβ1, with and without Y-27632, were seeded in type I collagen gels. Contraction of the gels was then monitored over three days, which disclosed that TGFβ1 induced a significant contraction of fibroblast-seeded collagen gels (Figure 3). The application of Y-27632 along with TGFβ1, however, almost totally negated this effect.

**Figure 4:**
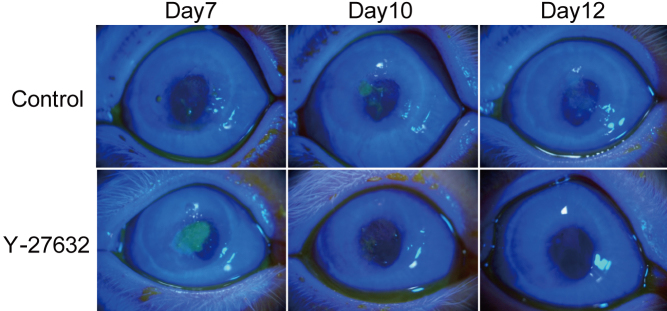
Macroscopic findings in the anterior segment. Epithelial wound closed at 5 days in vehicle-treated group after surgery, but it took 7–10 days in Y-27632 treated group.

To investigate whether Y-27632 could influence the fibroblastic transition and wound healing processes in vivo, a superficial wound in rabbits was created by the removal of a disc of anterior cornea 7.5 mm in diameter, comprising the epithelium and superficial stroma. Age-matched rabbit corneas were about 392±12 µm thick (mean±SE; n=4) when measured by ultrasonic pachymetry. After our surgeries, the average thickness of all eight operated corneas was 286±18 µm (mean±SE). This increased for 24 h after surgery, and then gradually reduced toward the initial thickness as epithelial healing progressed. Average corneal thickness in the vehicle-treated and Y-27632-treated groups at the three weeks postoperation showed no significant difference (p=0.524) at 354±17 µm and 378±31 µm, respectively. Healing corneas showed some haze in both vehicle and Y-27632 treated groups throughout the recovery period. The epithelial wound closed five days after surgery in the vehicle-treated group, but not until post-operative days 7 to 10 in the Y-27632 treated group (Figure 4), suggesting that Y-27632 causes a delay in epithelial cell migration and differentiation, which leads to a retarded resurfacing of the cornea wound surface.

**Figure 5:**
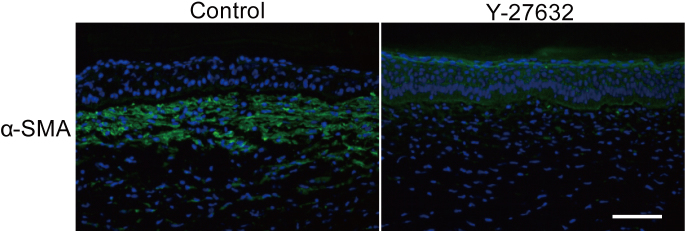
The effect of Y-27632 on keratocytes after superficial keratectomy. Y-27632 suppressed the expression of α-SMA at the center of the wound. Scale bar: 100 μm.

**Figure 6:**
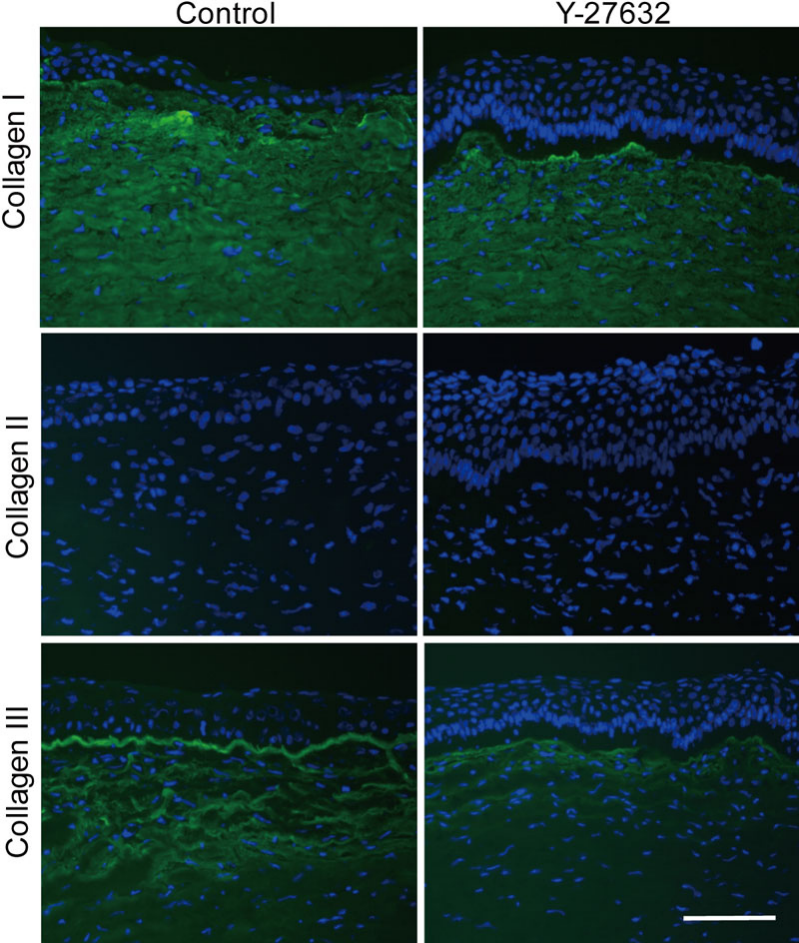
The effect of Y-27632 on collagen after superficial keratectomy. The level of collagen type I was unchanged and collagen type II was negative in both vehicle-treated and Y-27632 treated groups. Collagen type III signal, which was seen in the subepithelial layer in the center of vehicle-treated corneas, was more diffuse in Y-27632 treated corneas. Scale bar: 100 μm.

**Figure 7:**
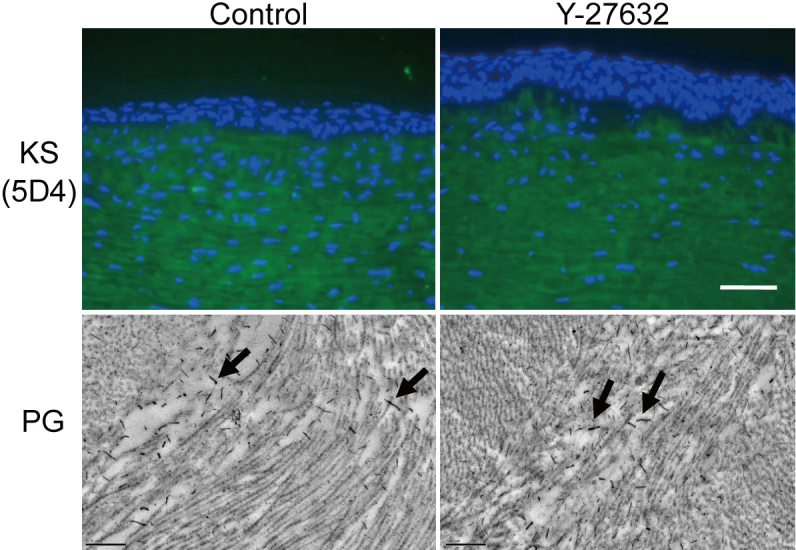
The effect of Y-27632 on proteoglycans after superficial keratectomy. No differences in KS-GAG distribution were evident from immunohistochemistry with the 5D4 antibody. Electron microscopy revealed that large cuprolinic blue-stained proteoglycan filaments, typical of healing stromal scars and presumably of the chondroitin sulfate/ dermatan sulfate subfamily, were present in both groups. Scale bar: top; 100 μm, below; 0.5 μm.

**Figure 8:**
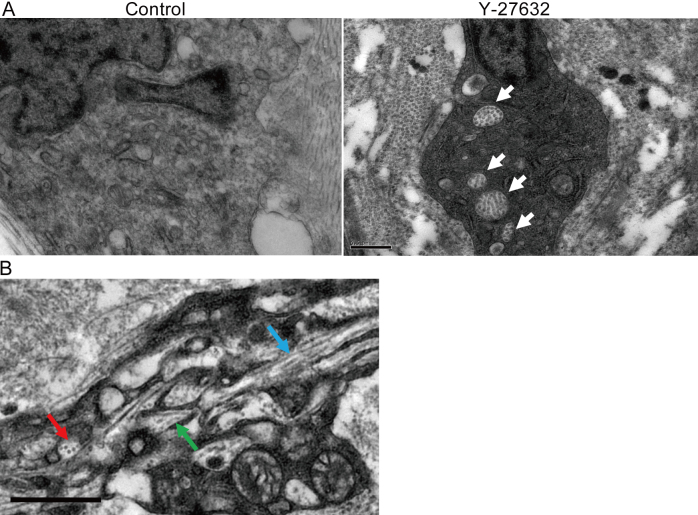
The effect of Y-27632 on the prevalence of fibripositors. **A**: Bundles of aligned and uniformly spaced collagen fibrils (arrows) were more prevalent in keratocytes in Y-27632 treated corneas. These resembled fibripositor-like structures, which have been proposed in tendon as a mechanism of uniaxial matrix deposition. These tend to be features of developing, rather than mature, connective tissue matrices. **B**: Importantly, keratocytes seem to show multiple fibripositor directions. (Arrows; blue=longitudinal, red=transverse, green=oblique) Scale bar: **A**; 0.5 μm, **B**; 1 μm.

Immunohistochemical investigations of corneas three weeks post-operation showed that Y-27632 suppressed α-SMA at the center of the wound (Figure 5), which was consistent with in vitro data. At the wound edge, however, α-SMA expression was evident in both vehicle-treated and Y-27632-treated groups. With regard to matrix synthesis in the healing cornea, we noted that collagen type I, the major component of the corneal stroma, was present and unchanged three weeks after surgery in both vehicle-treated and Y-27632-treated corneas. Collagen type II, a component of embryonic corneal tissues, was absent in both vehicle-treated and Y-27632-treated groups (Figure 6), although the antibody gave a strong signal in cartilage tissue from rabbit ears used as a positive control (data not shown). Collagen type III signal, however, which is characteristic of corneal scar tissue, was positive in the subepithelial stroma in the center of vehicle-treated corneas, but diminished in Y-27632 treated tissue (Figure 6). No changes in the distribution of sulfated keratan sulfate glycosaminoglycans were evident from immunohistochemistry with the 5D4 antibody in vehicle-treated and Y-27632-treated corneas (Figure 7). Moreover, electron microscopy revealed that large proteoglycan filaments, typical of healing corneal scar tissue, were present equally in both groups (Figure 7). The topical application for three weeks of Y-27632 eye drops resulted in the appearance of keratocytes that contained bundles of from 5 to 30 highly-aligned and uniformly-spaced collagen fibrils (Figure 8). These structures, which are common features of embryonic cornea (data not shown), were not seen in the vehicle-treated tissue.

## Discussion

An understanding of cell behavior in repair mechanisms following wounding is imperative if we are to modulate phenotypic transitions and avoid excessive scarring in healing tissues. Cytokines and growth factors are undoubtedly influential in this regard, a point emphasized by the fact that the expression patterns of these molecules in the fetus, which has the ability to heal by scarless regeneration, are unlike those in the adult, where scarring invariably occurs. Thus, scarless healing may be influenced by cytokines and growth factors that direct cell differentiation. Accordingly, work by Sullivan and associates [40] showed that TGFβ is present in adult human skin, which heals with the formation of a scar, but not in scar-free wounds in fetal human skin. Shah and coworkers [41] highlighted the involvement of TGFβ in scar formation, and showed that dermal wounds in adult rats treated with a neutralizing antibody to TGFβ healed without scarring. In previous work, we also identified that the TGFβ1 receptor inhibitor (SB431542) inhibited the excessive transformation of keratocytes in vitro, as evaluated by immunohistochemistry for α-SMA, and restricted scarring in vivo when it was injected into the rabbit cornea along with TGFβ1 (data not shown). Thus, the relative lack of TGFβ has been proposed as one mechanism whereby fetal tissues may regenerate by scarless healing. Here, we confirm the TGFβ1-induced differentiation of keratocytes into myofibroblasts in vitro—evidenced by the spindle-like cell morphology and increased levels of cell-associated α-SMA—and investigate the possible effects of a selective ROCK inhibitor, Y-27632, on the modification of this cellular transition in vitro and in vivo.

As reported previously, TGFβ1 induces a contraction of fibroblast-seeded collagen gels via myofibroblast transition, which is possibly aided by the downstream involvement of connective tissue growth factor [42,43]. Our data also demonstrate a functional change in keratocytes seeded in collagen gels in the presence of TGFβ1, which results in the contraction of untethered gels, and further demonstrates that this activity is abolished in cell-seeded collagen gels if Y-27632 is present. Thus, the inhibition of TGFβ1-induced myofibroblast transformation by Y-27632 at the cellular level in culture translates to an effect on function at the tissue level through the abolition of cell-mediated modulation of a simple matrix.

The inhibition of TGFβ1-induced α-SMA by the action of Y-27632, which was seen in vitro by us and by others [27] is also evident in vivo, with stromal cells in the center of Y-27632-treated corneas showing no immunohistochemical expression of α-SMA three weeks after wounding, unlike the corneas of vehicle-treated controls. A key role for cells in healing tissue is to synthesis new tissue, and a host of studies has shown that the transformation of keratocytes is involved in extracellular matrix changes. Central areas of healing corneas examined in the present study displayed normal levels of collagen type I throughout the observation period irrespective of treatment. In contrast, the elevated signal for collagen type III, which was seen in vehicle-treated controls, was absent when Y-27632 was used. Collagen type III has been reported as a minor component of mature (human) cornea [44,45], and published evidence has pointed to its upregulation in scar tissue [46-49]. Thus, the absence of collagen type III signal following Y-27632 treatment, compared with vehicle-only treatment, could be an indicator of a less aggressive type of tissue regeneration in the presence of Y-27632. It was recently reported that cell-associated keratan sulfate was reduced in keratocytes that had differentiated into myofibroblasts in vitro under the influence of TGFβ1, and that this change was minimized in the presence of Y-27632 [27]. For the keratan sulfate core proteins, lumican and keratocan, mRNA was also shown to be between 60% and 79% lower following TGFβ1-induced cellular differentiation in vitro, with Y-27632 negating the reduction in lumican, but not in keratocan [27]. Our in vivo studies revealed no change in sulfated keratan sulfate three weeks after wounding, with or without Y-27632 application, as identified by immunohistochemistry with an antibody (5D4), which recognizes a minimally pentasulfated epitope on the keratan sulfate glycosaminoglycan chain [50]. Large proteoglycan filaments, which are seen in the stromal matrix in the center of the healing wound, are similar in character to stained structures that have been reported previously in healing corneas [34,35,49] and in embryonic corneas [51]. In these tissues, they represent oversulfated proteoglycans of the chondroitin sulfate/dermatan sulfate class [52], and it is likely that that is the case here too, although we did not definitively identify the glycosaminoglycan as chondroitin sulfate/dermatan sulfate by prior lyase digestion. Their presence was not diminished by the topical application of Y-27632, which indicates a lack of impact on the glycosaminoglycan biosynthetic pathway in the healing tissue.

In vivo healing of superficial corneal wounds by the inward migration of epithelial cells from the stem cell niche at the edge of the cornea [53] was delayed by Y-27632 treatment. Full epithelial coverage was achieved five days after surgery in vehicle-treated eyes, but took 7 to 10 days in the Y-27632 treated group. This result is likely related to changes in the cell cycle, based on reports that Y-27632 downregulates the assembly of E-cadherin and connexion-43 cell-cell junctions in corneal epithelial cells and causes a delay in the G_1_/S cell cycle progression [54-56]. It is possible that this retarded epithelial coverage, which, as mentioned, proceeds inwardly from the corneal periphery, will influence some of the differences seen between the wound edge and center. Cell communication between the epithelium and stroma is believed to be important in corneal homeostasis and wound healing, and Wilson and associates [57] proposed that epithelium-derived cytokines stimulate mitosis and chemotaxis of myofibroblasts, and that myofibroblast-derived cytokines stimulate epithelial cell proliferation and migration during wound healing. It has been reported that TGFβ1 is produced by the corneal epithelium [15,16,57]. Consequently, our finding that Y-27632 suppressed α-SMA expression at the center of the cornea, but not at the edge, three weeks after surgery might be the result of a simple competitive balance between the agents (i.e., TGFβ1 and Y-27632 at the concentration and frequency used), with prolonged exposure to TGFβ1 at the wound edge from earlier wound healing stages.

Detailed electron microscopy examination of the wound center of Y-27632-treated corneas three weeks after surgery revealed the presence of numerous cellular inclusions containing bundles of uniform diameter and equally spaced collagen fibrils. These are not seen in vehicle-treated corneas. Interestingly, the cellular inclusions in cornea treated with Y-27632 resemble fibripositor-like structures, which have been proposed in embryonic tendon as a mechanism of uniaxial matrix deposition [58]. In this concept of matrix deposition based on developing tendon, fibripositors (or fibril depositors) are Golgi-to-plasma membrane carriers containing procollagen, which, upon secretion into the extracellular matrix, is cleaved to initiate collagen fibril formation. In this way, collagen fibrils are extruded from the plasma membrane and delivered into the extracellular space, where they are often aligned laterally with other extruded fibrils. Bundles of laterally organized collagen fibrils have also been documented in small membrane invaginations at the edge of keratocytes in embryonic chick corneas, suggesting that collagen fibrillogenesis occurs in small surface recesses [59]. Regardless of the precise mechanism of matrix deposition in connective tissue development, it is notable that embryonic cells prefer to lay down collagen fibrils in well arranged bundles, rather than in a disorganized mass of fibrotic scar tissue. Cells in the centers of corneal wounds treated with Y-27632, unlike those treated with vehicle only, displayed bundles of aligned collagen fibrils that were regularly spaced and of uniform diameter, which resembled the features reported in embryonic connective tissue matrices of tendon and cornea [58,59]. However, widespread matrix changes and an increase in the characteristic embryonic collagen sub-type, type II, were not seen at the level of immunohistochemistry. Interestingly, the keratocytes in our Y-27632-treated healing corneas contained collagen fibril bundles within the cellular inclusions, which are oriented in multiple directions—longitudinal, transverse, and oblique to the section plane—thus mimicking the formative architecture of the corneal stroma. These observations suggest that Y-27632, by inhibiting the transition of keratocytes into myofibroblasts, might cause cells in the healing adult rabbit cornea to take on a partial embryonic character instead of the character of a typical myofibroblast, thus avoiding scar tissue formation in preference to an ordered regeneration of the wounded tissue.
